# The Central Conserved Peptides of Respiratory Syncytial Virus G Protein Enhance the Immune Response to the RSV F Protein in an Adenovirus Vector Vaccine Candidate

**DOI:** 10.3390/vaccines12070807

**Published:** 2024-07-20

**Authors:** Pengdi Chai, Yi Shi, Junjie Yu, Xiafei Liu, Dongwei Li, Jinsong Li, Lili Li, Dandi Li, Zhaojun Duan

**Affiliations:** 1 National Key Laboratory of Intelligent Tracking and Forecasting for Infectious Diseases (NITFID), NHC Key Laboratory for Medical Virology and Viral Diseases, National Institute for Viral Disease Control and Prevention, Chinese Center for Disease Control and Prevention, Beijing 102206, Chinal1420339660@163.com (D.L.);; 2 College of Public Health, Gansu University of Chinese Medicine, Lanzhou 730101, China; syys1568@163.com (Y.S.);; 3 The First Clinical Medical Institute, Henan University of Chinese Medicine, Zhengzhou 450046, China

**Keywords:** RSV vaccine, adenovirus vector, central conserved peptides of G protein, cis-regulatory motif, mucosal immunity

## Abstract

Respiratory syncytial virus (RSV) is a serious human respiratory pathogen that commonly affects children, older adults, and immunocompromised individuals. At present, the design of licensed vaccines focuses on the incorporation of the pre-fusion protein (PreF protein) of RSV, as this protein has the ability to induce antibodies that offer a high level of protection. Moreover, the G protein contains the CX3C motif that binds the chemokine receptor CX3CR1 in respiratory epithelial cells, which plays an essential role in viral infection. Therefore, incorporating the G antigen into vaccine design may prove more advantageous for RSV prevention. In this study, we developed a human adenoviral vector-based RSV vaccine containing highly neutralizing immunogens, a modified full-length PreF protein fused with the central conserved peptides of the G protein (Gcc) from both RSV subgroups trimerized via a C-terminal foldon, and evaluated its immune response in mice through intranasal (i.n.) immunization. Our results showed that immunization with Ad5-PreF-Qa-Gcc elicited a balanced Th1/Th2 immune response and robust mucosal immunity with higher neutralizing antibody titers against RSV Long and RSV B1. Importantly, immunization with Ad5-PreF-Qa-Gcc enhanced CD4^+^ CD25^+^ FoxP3^+^ Treg cell response and protected the mice against RSV infection. Our data demonstrate that the combination of Gcc and the PreF antigen is a viable strategy for developing effective RSV vaccines.

## 1. Introduction

Respiratory syncytial virus (RSV) is the most common cause of acute respiratory infection in infants, older adults, and immunocompromised individuals [1]. In 2019, nearly 32 million children under 5 years of age were infected by RSV worldwide, which could develop into a lower respiratory tract infection, with 10% requiring hospitalization, making them prone to the subsequent development of obstructive airway diseases and asthma [2]. Moreover, individuals impacted by RSV infection fail to elicit durable antibody responses, resulting in reinfection being a common occurrence. Consequently, this disease poses a substantial medical and economic burden globally.

At present, two recombinant protein vaccines have been authorized and approved, and several vaccines have been the subject of clinical studies, in which efforts have been predominately focused on the PreF antigen. The F protein is a type I membrane protein, conserved between the RSV A and RSV B antigenic subgroups, and it exists in at least two conformations: a metastable conformation, PreF, and a stable conformation, PostF. PreF contains the unique antigenic epitopes site Ø and site V, which induce almost 90% of the F-specific neutralization antibodies in humans compared to PostF. However, notable changes in the antigenic sites of strain A2 and strain B compared to the epidemic strain were observed in 2013. In particular, a higher-frequency polymorphism was observed at site Ø (I206M/Q209R) and site V (L172Q/S173L/K191R) of the F protein in strain B, which may be associated with vaccine escape mutations against the F protein and monoclonal antibody [3]. Additionally, anti-G antibody levels in adults have been found to show a significant decrease, which may be a factor in sustained susceptibility to RSV infections throughout life [4]. It is necessary to increase the diversity of antigens utilized to improve the protective efficacy of RSV vaccines. RSV G is a type II membrane protein composed of a central conserved domain (Gcc) consisting of approximately 40 highly conserved amino acids and two mucin-like regions that exhibit high variability. Due to its high glycosylation rate and ability to induce pulmonary eosinophilia and immune escape, as demonstrated in a mouse model, caution must be exercised when considering the use of full-length G protein or secreted G protein (sG) [5,6,7]. However, the bacterially produced unglycosylated G protein cannot induce vaccine-enhanced disease (VED) and showed suitable safety and immunogenicity in phase II of a clinical trial [5]. These factors limit the application of the full-length G protein in vector vaccines. Interestingly, Gcc shows no glycosylation and is highly conserved in subgroups A and B, which contain the CX3C motif that binds to the chemokine receptor CX3CR1 in human respiratory epithelial cells and plays an important role in viral infection and virus pathogenesis [8,9]. Incorporating an immunogen targeting the RSV Gcc immunogen may enhance effectiveness and universality while also reducing escape potential over an RSV F-alone approach.

In this study, we developed a serotype 5 human adenoviral vector (Ad5)-based RSV vaccine carrying PreF and Gcc linked through the self-cleaving peptide P2A. Additionally, we introduced a novel unique 21-mer cis-regulatory motif (Exin21) referred to as Qa, which has been reported to enhance protein expression/secretion via mRNA stabilization [10]. We showed that the PreF-Qa-Gcc antigen design strategy based on the Ad5 vector vaccine platform induced both mucosal and systemic immune responses, effectively safeguarding against RSV challenge. In particular, the incorporation of Gcc into the vaccine design enhanced the immune homeostasis of Treg cells in mice after challenge.

## 2. Materials and Methods

### 2.1. Cells and Virus

The A549, Hep2, and HEK293A cells used in this study were all cultured in Dulbecco’s modified Eagle’s medium (DMEM, Gibco, USA) supplemented with 10% fetal bovine serum (FBS, Gibco, Grand Island, NY, USA), 100 IU of penicillin, and 100 μg/mL streptomycin (Gibco, USA) at 37 °C and 5% CO_2_. The RSV Long strain (VR-26) was obtained from the Chinese Center for Disease Control and Prevention. The RSV B1 strain (VR-26) was obtained from Vazyme. The RSV Long and RSV B1 strains were passaged in Hep-2 cells in DMEM/2%FBS, purified via ultrafiltration on a 35% sucrose cushion, and centrifuged at 15,000 rpm for 5 h at 4 °C using an S50A rotor (Hitachi, Tokyo, Japan) [11]. The virus pellets were re-suspended in MEM/3% sucrose and stored at −80 °C.

### 2.2. Vaccine Preparation

The full-length F gene of the RSV A CC14-10 (GenBank: APW77972) was modified by introducing amino acid substitutions (N67I, R106Q, F137S, S215P, and E487Q), and P27 (109–136aa) was substituted with the linker (GSGSGR) to generate a PreF construct [12]. The Gcc-foldon was prepared as described in a previous study [13]; a dimer of Gcc peptides from strains A and B was fused together and followed C-terminally with a T4 foldon. Ad-PreF-Gcc was encoded by separating the PreF and Gcc-foldon with a P2A peptide, driven by a cytomegalovirus promoter, and lastly cloned onto the E1 region of Ad5-empty using Gibson assembly. Ad-PreF-Qa-Gcc, a 21-mer cis-regulatory motif (CAACCGCGGTTCGCGGCCGCT, Qa), was inserted in the front of P2A preceding the PreF C terminal of Ad-PreF-Gcc. In addition, we constructed Ad-PreF and Ad-Gcc encoding the PreF/Gcc-foldon as controls. Recombinant adenoviruses were recovered and propagated in HEK293A cells and purified via CsCl gradient ultracentrifugation as described in a previous study [14]. The infectious units (IFUs) were titrated on HEK293A cells using an Adeno-X Rapid Titer Kit (Takara, Tokyo, Japan) following the manufacturer’s instructions. The FI-RSV vaccine was prepared according to the method detailed in a previous study [15].

### 2.3. Western Blot

The HEK293A cells were separately transduced with 10^8^ IFUs of Ad-PreF, Ad-Gcc, Ad-PreF-Gcc, and Ad-PreF-Qa-Gcc. At 24 h post-infection, the cells lysed by the RIPA lysis buffer (Beyotime, Shanghai, China) and supernatant were collected for SDS-PAGE and then transferred onto PVDF membranes for Western blot analysis using a mouse anti-F mAb clone 133-1H (1:1000, Merck, Darmstadt, Germany) or a mouse anti-G mAb clone131-G (1:1000, Abcam, USA), which was followed by the use of an HRP-conjugated goat anti-mouse IgG antibody (1:5000, Abcam, Waltham, MA, USA). GAPDH was selected as the internal control (Proteintech, Wuhan, China).

### 2.4. Characterization of RSV PreF

To identify the conformation of the PreF protein, the A549 cells were separately transduced with Ad5-PreF and Ad5-PreF-Gcc at MOI = 250. The Ad5-empty-transduced cells were used as controls. At 24 h post infection, the cells were digested by trypsin into a single-cell suspension. Next, 1 × 10^6^ cells were stained with F488 Anti-Respiratory Syncytial Virus Fusion Protein Antibody (Φ) (1:100, Vazyme, Nanjing, China). Afterward, the data were acquired using a BD Arial II and analyzed using Flowjo 10.

### 2.5. Immunization and Challenge of Mice

In this study, 6- to 8-week-old female BALB/c mice were purchased from Beijing Vital River Laboratory Animal Technology Co., (Beijing, China). The Balb/C mice were separately intranasally (i.n.) and inoculated with a single dose of 2 × 10^8^ IFU Ad5-empty, Ad5-PreF, Ad5-Gcc, or Ad5-PreF-Qa-Gcc. For the FI-RSV control, the mice were immunized i.m. with 20 μg of FI-RSV using a prime–boost immunization strategy at weeks 0 and 2. Blood was collected from the mice at 2-week intervals after immunization. The mice were euthanized 8 weeks after prime immunization through carbon dioxide inhalation. Bronchoalveolar lavage fluid (BALF) was collected for subsequent assays by inserting an 18-gauge needle into the trachea and washing 1 mL of PBS plus 1% bovine serum albumin (BSA) into and out of the lung with the BALF being stored at −80 °C.

To evaluate the protective efficacy of the vaccine candidates against RSV, the immunized BALB/c mice were i.n. challenged with 2 × 10^6^ PFU RSV Long at week 6. Five days post-challenge, the mice were euthanized through carbon dioxide inhalation, and their lung tissue was collected for subsequent assays. The right cranial lobe of the lung was immersed in 4% paraformaldehyde. The other tissue was quickly frozen with liquid nitrogen and stored at −80 °C.

### 2.6. Antibody ELISAs

PreF, G-specific IgG, IgG1, IgG2a, and IgA antibody levels in the mouse sera and BALF were measured using ELISA, as described in a previous study [14]. Briefly, 96-well ELISA plates (Corning, Shanghai, China) were coated overnight at 4 °C with 75 ng/well of purified PreF or G in Na_2_CO_3_-NaHCO_3_ buffer (pH9.6). After blocking using 5% skim milk at 37 °C for 2 h, serial dilutions of the mouse sera in PBS/Tween-20 (PBST) containing 2% skim milk were added to the wells and incubated at 37 °C for 1 h. The HRP-conjugated goat anti-mouse IgG (1:30,000), IgA (1:5000), IgG1 (1:20,000), or IgG2a (1:20,000) (Abcam, USA) was used for detection for 1 h at 37 °C. Following three washes with PBST, TMB was added for 10 min; then, the reaction was stopped with 2 M H_2_SO_4_, and absorbance was measured at 450 nm and 630 nm using a microplate reader. The endpoint titer was determined as the reciprocal of the highest serum dilution that yielded an absorbance greater or equal to 2.1 times the absorbance of the negative samples.

### 2.7. Neutralization Assay

A microneutralization assay was used to determine the anti-RSV Long and RSV B1 neutralizing antibody titer, following the method described in a previous study [16]. Briefly, mouse heat-inactivated serum was threefold diluted with an initial dilution of 1:20 and mixed with an equal volume of 100 pfu RSV Long or B1 and then incubated at 37 °C for 2 h; afterward, 50 μL DMEM (2% FBS, 100 units/mL penicillin/streptomycin) containing 2 × 10^4^ A549 cells was added per well and incubated for 72 h at 37 °C. The cells were fixed with 80% acetone, and after three washes with PBS, the mouse anti-RSV antibody clone 133-1H (Sigma, Steinheim, Germany) was diluted 1:1000 and added to the ELISA plates for an additional 1 h of incubation. After three washes with PBST (PBS + 0.05%Tween 20), HRP-conjugated goat anti-mouse IgG (1:10,000, Abcam, USA) was used for detection for 1 h at 37 °C. After three washes with PBST, TMB (Solarbio, Beijing, China) was added for 10 min; then, the reaction was stopped with 2 M H_2_SO_4_, and absorbance was measured at 450 nm using a microplate reader. The neutralization titers were defined as the reciprocal of the serum dilution required for 50% neutralization of viral infection.

### 2.8. Histological Analysis

To perform the histological analysis, the whole lungs were harvested on day 5 following RSV challenge, and the right cranial lobe of the lung was immersed in 4% paraformaldehyde. After being embedded and sectioned, the tissues were stained with hematoxylin and eosin (H&E) and periodic acid–Schiff (PAS) for pathological evaluation and observation of mucus secretion.

### 2.9. Treg Cells and Th17 Cells

To isolate lung lymphocytes from the mice, the lungs were cut into pieces and digested in 2 mL of PBS buffer containing 1.5 mg/mL collagenase D (Roche, Basel, Switzerland) and 0.2 mg/mL DNase I (Roche, Switzerland) for 30 min at 37 °C. Following digestion, the lung was pressed through a 40 μm nylon mesh screen. After being collected and washed, the cells were aspirated from the discontinuous Percoll gradients of 40% and 80%. The lymphocytes were blocked with the anti-CD16/CD32 monoclonal antibody (1:300, BD Biosciences, San Jose, CA, USA) and stained using a Live/Dead Stain Kit (1:1000, Thermo, Waltham, MA, USA) in DPBS. Afterward, the cells were surface-stained with FITC-CD4 (1:400, BD Biosciences, Cat#553047) and BV421-CD25 (1:300, BD Biosciences, Cat#562606). Following fixation and permeabilization, the cells were intracellularly stained with AF647-Foxp3 (1:150, BD Biosciences, Cat#560401) and PE-IL-17A (1:300, BD Biosciences, Cat#559502). Lastly, the samples were analyzed using a BD Arial II (BD Biosciences, USA).

### 2.10. Quantitative RT-PCR Assay

The RSV load in the lung and nasal tissues was quantified using q-RT-PCR, and viral RAN was extracted using a FastPure Viral DNA/RNA Mini Kit (Vazyme, China) according to the manufacturer’s instructions. RSV L gene copies were quantified using a HiScript II One Step qRT-PCR Probe Kit (Vazyme, China) in a Gentier 96E/96R (TIANLONG, Xi’an China) and calculated with a standard curve. The qRT-PCR primer sequences are listed in Table 1.

### 2.11. Statistical Analysis

All data are presented as the mean ± SEM. Statistical analyses were performed with GraphPad Prism 9. Comparisons of various groups were undertaken using analysis of variance (ANOVA) followed by Tukey’s test. Asterisks in the figures indicate the level of statistical significance (* *p* < 0.05, ** *p* < 0.01, *** *p* < 0.001, and **** *p* < 0.0001).

## 3. Results

### 3.1. A Heptapeptide, Qa, Can Boost Protein Expression and Improve Self-Cleaving Efficiency

**Figure 1:**
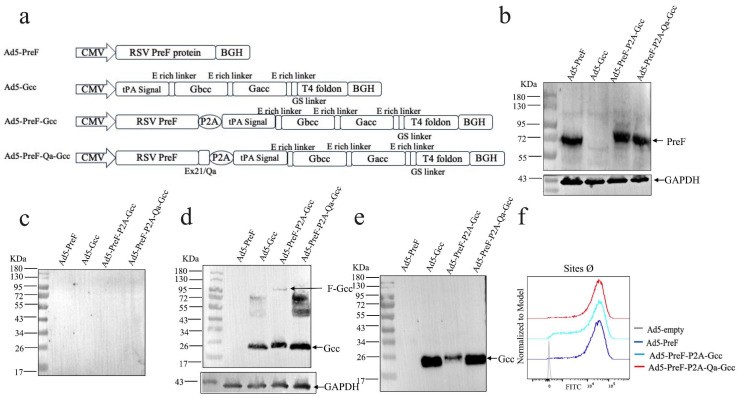
Designing and characterizing the Ad-based vaccines. (**a**) Schematic of Ad5-PreF, Ad5-Gcc, Ad5-PreF-P2A-Gcc and Ad5-PreF-Qa-P2A-Gcc. (**b**,**c**) Western blot analysis of F expression in cell lysis (**b**) and supernatant (**c**). (**d**,**e**) Western blot analysis of Gcc expression in cell lysis (**d**) and supernatant (**e**). (**f**) Identification of the conformation of F protein. Ad5-empty was used as control. Site Ø represents the unique antigenic sites of PreF.

In the present study, we fused PreF and Gcc using the self-cleaving peptide P2A (Figure 1a). The results of our Western blot analyses confirmed the expression of F proteins only in the lysates and Gcc protein in both the supernatant and lysates of HEK293A cells (Figure 1b–e). However, the expression of the Gcc protein in Ad5-PreF-Gcc was notably reduced with some fusion proteins remaining uncleaved in vitro. In contrast, the expression level of Gcc in Ad5-PreF-Qa-Gcc was significantly enhanced compared with Ad5-F-Gcc, reaching a level comparable to that of Ad5-Gcc. The expression level of preF was similar to that of Ad5-F and Ad5-F-Gcc (Figure 1b). Surprisingly, the cleavage efficiency of P2A reached 100% (Figure 1d), suggesting that Qa could boost Gcc protein expression and improve self-cleaving efficiency. Furthermore, we also examined whether the addition of P2A and Gcc would affect the confirmation of PreF. The results showed that Ad5-PreF, Ad5-PreF-Gcc, and Ad5-PreF-Qa-Gcc could bind to antibodies against pre-fusion conformational monomers (Figure 1f), suggesting that the addition of P2A and Gcc does not affect the PreF conformation at critical antigenic sites.

### 3.2. Intranasal Immunization with Ad5-PreF-Qa-Gcc Induces a High-Level Antibody Response in Mice

**Figure 2:**
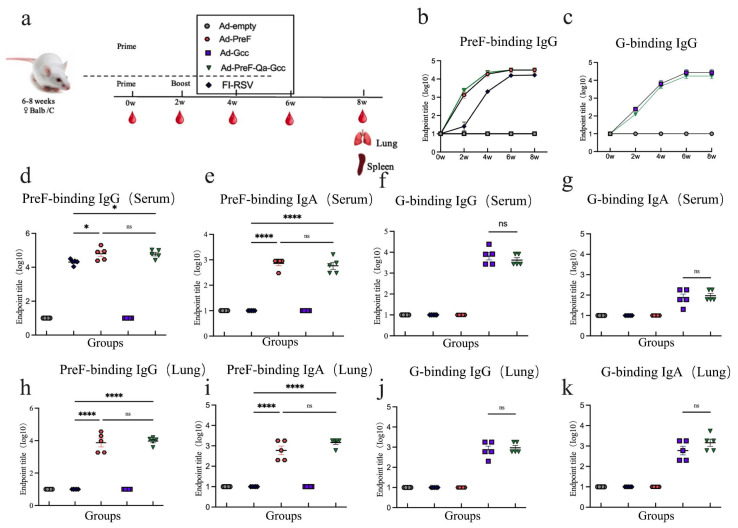
Immune responses in immunized Balc/c mice. (**a**) Schedule of Ad5 vaccines immunization and bleeding strategies in female Balb/C mice. (**b**) Kinetics of PreF-specific total IgG reciprocal endpoint titers (log10) were measured within 8 weeks of initial vaccination. (**c**) Kinetics of G-specific total IgG reciprocal endpoint titers (log10) were measured within 8 weeks after vaccination. (**d**) Endpoint titer of PreF-specific total IgG was measured in serum at week 6. (**e**) Endpoint titer of PreF-specific total IgA was measured in serum at week 6. (**f**) Endpoint titer of G-specific total IgG was measured in serum at week 6. (**g**) Endpoint titer of G-specific total IgA was measured in serum at week 6. (**h**) Endpoint titer of PreF-specific total IgG was measured in BALF at week 8. (**i**) Endpoint titer of PreF-specific total IgA was measured in BALF at week 8. (**j**) Endpoint titer of G-specific total IgG was measured in BALF at week 8. (**k**) Endpoint titer of G-specific total IgA was measured in BALF at week 8. ns = not significant; * *p* < 0.05, **** *p* < 0.0001.

PreF-specific IgG and G-specific IgG were induced in a time-dependent manner, reaching their highest level at week 6 compared to the observations made in the control mice (Figure 2b,c). Ad-PreF-Qa-Gcc induced a higher PreF-binding IgG titer in serum at 6 w (Figure 2d) compared to the mice immunized with FI-RSV, which was similar to the results noted for the mice vaccinated with Ad5-PreF. No significant difference was observed between the Ad5-Pre and Ad-PreF-Qa-Gcc groups (Figure 2d). Regarding anti-G antibody levels, mice immunized with Ad5-PreF-Qa-Gcc showed similar results to those immunized with Ad5-Gcc (Figure 2f). We also evaluated the antigen-specific IgA antibody in the BALF and serum of the mice and found that FI-RSV did not induce a response in either the sera or the BALF (Figure 2e,i). In contrast, high antigen-specific IgG and IgA antibody titers were observed in both the sera and BALF of the mice immunized with Ad5-PreF, Ad5-Gcc, and Ad5-PreF-Qa-Gcc (Figure 2g–i,k).

**Figure 3:**
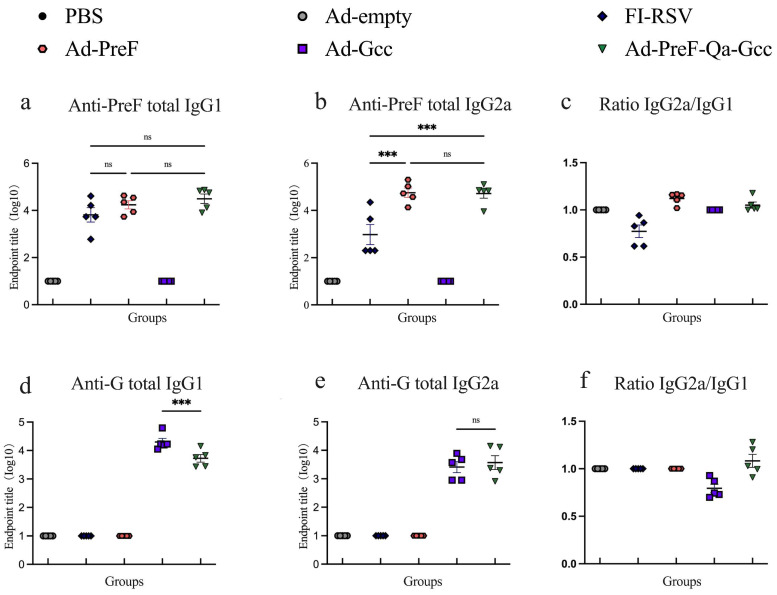
Intranasal immunization induced a balanced Th1/Th2 immune response in mice vaccinated with Ad-PreF-Qa-P2A-Gcc. (**a**–**c**) Anti-PreF antibody subtype IgG1 (**a**) and IgG2a (**b**) were detected in serum at week 6, and the ratio of IgG2a/IgG1 (**c**) was calculated. (**d**–**f**) Anti-G antibody subtypes IgG1 (**d**) and IgG2a (**e**) were detected in serum at week 6, and the ratio of IgG2a/IgG1 (**f**) was calculated. ns = not significant; *** *p* < 0.001.

We also assessed the antigen-specific IgG1 and IgG2a antibody titers, as well as the ratio of IgG2a/IgG1 in the vaccinated mice at week 6. In comparison to the results for FI-RSV, vaccination with Ad5-PreF and Ad5-PreF-Qa-Gcc induced a higher anti-PreF IgG2a titer; however, a similar IgG1 antibody titer was noted (Figure 3a,b). Ad5-PreF-Qa-Gcc elicited a balanced Th1/Th2 response with median IgG2a/IgG1 ratios of 1.051. In contrast, immunization with FI-RSV induced a Th2-biased response with an IgG2a/IgG1 ratio of 0.7735 (Figure 3c). Interestingly, mice immunized with Ad5-PreF-Qa-Gcc induced similar anti-G IgG2a antibody levels and lower anti-G IgG1 antibody levels than the mice vaccinated with Ad5-Gcc (Figure 3d,e). Ad5-PreF-Qa-Gcc elicited a balanced Th1/Th2 response with an IgG2a/IgG1 ratio of 1.083 in the mice. However, Ad5-Gcc induced a Th2-biased response with an IgG2a/IgG1 ratio of 0.7941 (Figure 3f). Our data demonstrate that Ad5-PreF-Qa-Gcc elicited a balanced Th1/Th2 response and high mucosal immunity in the Balb/C mice.

### 3.3. The Addition of the Gcc Antigen Improved the Neutralizing Antibody Titers against RSV Long and RSV B1

**Figure 4:**
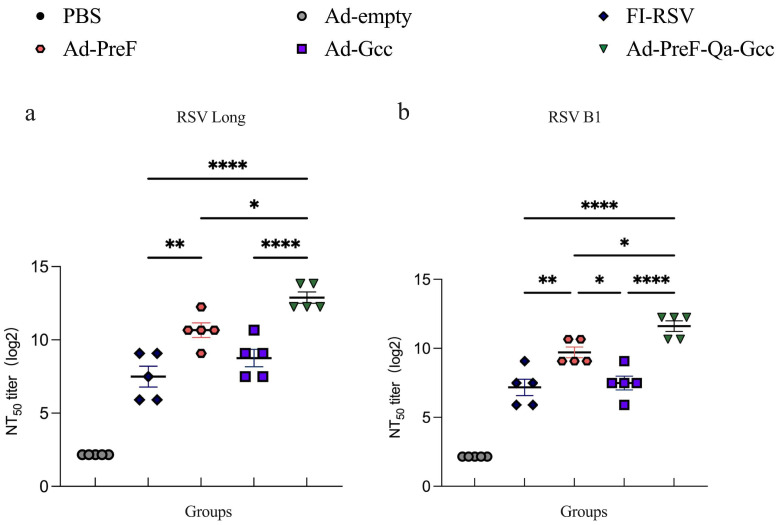
Mice vaccinated with Ad5-PreF-Qa-Gcc induced superior neutralizing antibody. (**a**,**b**) Serum-neutralizing antibody response against RSV Long (**a**) and RSV B1 (**b**) at week 6. All data are shown as means ± SEM. *p*-values were analyzed with one-way ANOVA (* *p* < 0.05; ** *p* < 0.01; **** *p* < 0.0001).

The results of the neutralizing antibody (NAb) test showed that all groups induced specific RSV Long and RSV B1 NAb except for the Ad5-empty group in the sera of the vaccinated mice (Figure 4). The NAb titers in the Ad-Gcc vaccinated mice were similar to those of the vaccinated FI-RSV group; in comparison, those of the other groups were higher. Single intranasal immunization with Ad5-PreF-Qa-Gcc resulted in high NAb titers against RSV Long and RSV B1 when compared to the results noted for Ad5-PreF. The NAb titers were on average 4.6-fold and 3.7-fold higher for Ad5-PreF-Qa-Gcc compared to Ad5-PreF (Figure 4a,b), suggesting that the combination of PreF and the Gcc antigen increased the NAb titers compared to PreF or Gcc alone.

### 3.4. Intranasal Immunization Provided Protection against RSV Long In Vivo

**Figure 5:**
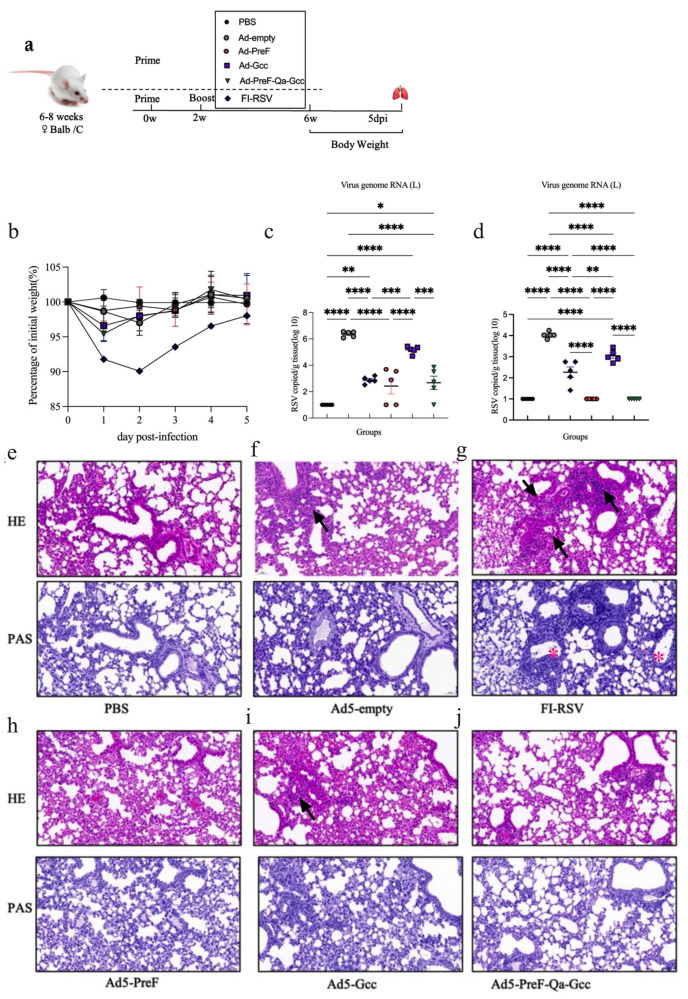
Immunogenicity and protective efficacy in BALB/c mice. (**a**) Schematics of vaccine immunization and challenge in BALB/c mice (6–8 weeks old). Mice were challenged with 2 × 10^6^ pfu RSV Long via the i.n. route at 4 weeks after boost vaccination. (**b**) Body weight change over 5 days post-infection. (**c**,**d**) Copies of RSV viral L gene in lung tissue (**c**) and nasal tissue (**d**). The limit of detection for the RT-qPCRs is 10 copies/mL. (**e**–**j**) Tissue sections of the lung tissues were stained with H&E and PAS for pathological examination. The magnification of all images is 20x. All data are shown as means ± SEM. *p*-values were analyzed with one-way ANOVA (* *p* < 0.05; ** *p* < 0.01; *** *p* < 0.001; **** *p* < 0.0001).

The process of immunization and challenge of RSV Long in Balb/C is shown in Figure 5a. Following challenge, the weight of the mice began to decrease on day 1 after challenge except for the PBS-challenged mice. However, mice vaccinated with FI-RSV not only lost the most weight (−90%) after challenge but also recovered more slowly than the mice in the other groups (Figure 5b). The RSV Long load in the lung and nasal tissues was quantified on day 5 post-challenge using qRT-PCR. As a control, the high virus copies in the lung (reaching roughly 10^6^ copies/g) were detected in the lungs of Ad5-empty-immunized mice, whereas very low RSV L gene copies (10^2^–10^3^ copies/g) were observed in the lungs of mice vaccinated with FI-RSV, Ad5-PreF, and Ad5-PreF-Qa-Gcc (Figure 5c). The same trend was also observed in the nasal tissue of the mice (Figure 5d). Interestingly, although the virus copies in the Ad5-Gcc-immunized mice decreased in both the lung and nasal tissues, there were no significant differences compared to the results of the mice vaccinated with Ad5-empty.

We further investigated pathological injury in the lungs. The results showed that the mice vaccinated with Ad5-empty and FI-RSV exhibited severe lung pathology, including moderately thickened alveolar walls and lymphocyte infiltration around the blood vessels and alveolar hemorrhage (Figure 5e). In contrast, the mice vaccinated with Ad5-PreF and Ad5-Gcc displayed signs of mild inflammation in the lungs. Importantly, the mice vaccinated with Ad5-PreF-Qa-Gcc presented similar histological features in their lungs compared to the PBS-treated mice. The severity of inflammation in the vaccinated mice was in the following order: FI-RSV > Ad5-empty > Ad5-Gcc > Ad5-PreF > Ad5-PreF-Qa-Gcc (Table 2). We also evaluated mucus secretion in the lungs using PAS staining. However, we only observed mild mucus secretion in the mice vaccinated with FI-RSV. No mucus secretion was observed in the other groups, whose results were similar to those of the PBS-challenged mice (Figure 5e).

### 3.5. PreF-Gcc Immunization Enhanced Treg Cells in the Post-Challenge Mice

**Figure 6:**
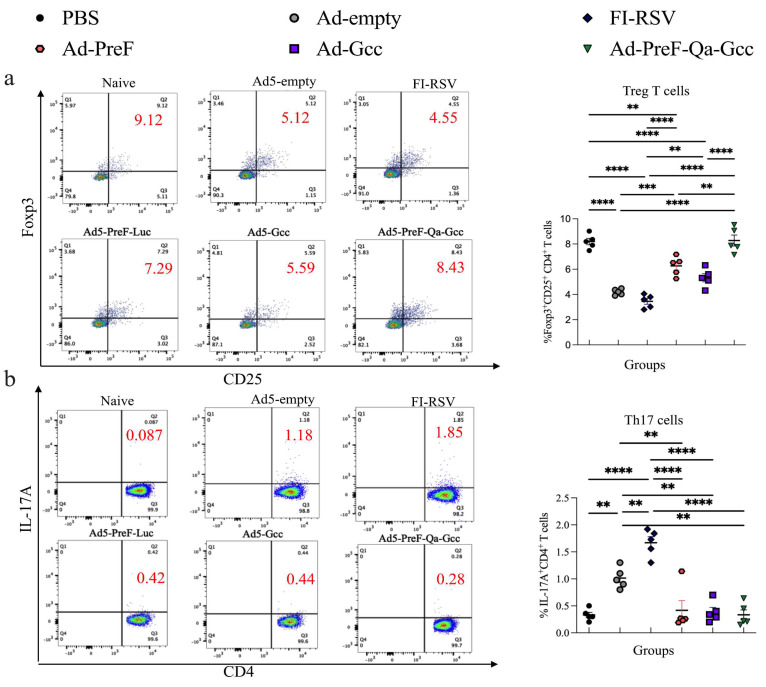
Treg cells and Th17 cells in the lung of vaccinated mice induced by RSV challenge infection. Vaccinated mice were challenged i.n. with RSV Long at 6 weeks after the immunization. (**a**) The percentage of CD4^+^CD25^+^Foxp3^+^ Treg cells. (**b**) The percentage of IL-17A^+^ CD4^+^ T cells. All data are shown as means ± SEM. *p*-values were analyzed with one-way ANOVA (** *p* < 0.01; *** *p* < 0.001; **** *p* < 0.0001).

In this study, we further examined CD4^+^ CD25^+^ FoxP3^+^ Treg cells and IL-17A^+^ CD4^+^ T cell subsets in the lungs of the vaccinated mice following RSV challenge. The data showed that the number of Treg cells in the FI-RSV-vaccinated mice significantly decreased compared with that in the PBS group not subjected to challenge (Figure 6a). Compared to the FI-RSV-vaccinated mice, the Treg cell levels of the mice vaccinated with Ad5-PreF and Ad-Gcc significantly increased. Interestingly, we found that the number of CD4^+^ CD25^+^ FoxP3^+^ Treg cells in the vaccinated Ad5-PreF-Qa-Gcc mice significantly increased compared to those vaccinated with Ad5-PreF or Ad5-Gcc, and no significant difference was noted compared to those challenged with PBS (Figure 6a). In contrast, significantly decreased IL-17A^+^ CD4^+^ T cell levels were observed in the mice vaccinated with Ad5-PreF, Ad5-Gcc, and Ad5-PreF-Qa-Gcc compared to the FI-RSV-immunized mice (Figure 6b), suggesting that the PreF-Gcc vaccine leads to a robust Treg cell response in the lungs of the challenged group that might eliminate the over-activation of lymphocytes and the inflammation caused by RSV infection.

## 4. Discussion

Vaccine development has been hampered primarily by the emergence of VED. It is widely believed that VED is related to Th2-biased immune response, poorly neutralizing antibodies, and CD4+ T cell subsets upon RSV infection [17]. Therefore, a safe and effective RSV vaccine should induce a balance of Th1-biased immune response and a high-affinity neutralizing antibody. Replication-deficient recombinant adenoviral vectors have numerous advantages over other viral vectors, including wide tissue tropism, inherent adjuvant properties, and the capacity to induce T cell immune response. To date, at least four RSV vaccines have been developed based on an adenoviral vector strategy, such as ChAd155, Ad26, Ad35, and Ad5. The presence of pre-existing immunity in the population poses a challenge for Ad5-based vaccines, necessitating strategies to mitigate its impact. Furthermore, reports on potential risks of thrombotic thrombocytopenia and disseminated intravascular coagulation associated with ChAdOx1 nCoV-19 and Ad26 COV2-S vaccines via an i.m. route against SARS-Cov2, particularly in young individuals, have raised concerns [18,19]. Respiratory mucosal delivery holds promise in addressing these immunity-related issues linked to the adenovirus vector itself and achieving an enhanced immune response [20]. Consequently, in our study, we selected Ad5 as the preferred adenovirus vector, and we advocated for intranasal administration as the optimal route of vaccine delivery.

Rainho-Tomko et al. reported that a nanoparticle vaccine based on RSV Gcc could elicit a potent Ab response in the native mouse model and that co-administering Gcc and PreF boosted humoral response without antigenic interference [13]. Furthermore, antibodies directed toward Gcc have been found to not only neutralize both RSV A and B in vitro but also reduce viral loads in animal models [21]. Thus, we combined PreF with Gcc in a specific antigen design using a P2A self-peptide. When expressed in vitro, we found that the expression of Gcc in Ad5-PreF-Gcc was decreased compared to that in Ad5-Gcc (Figure 1e). To address this issue, we introduced Qa sequences into the antigen design. Indeed, we found that the addition of Qa could significantly enhance the expression of Gcc. Surprisingly, it also improved 2A-mediated auto-cleaving efficiency (Figure 1e). Furthermore, we demonstrated that the addition of Qa-P2A-Gcc does not affect the conformation of PreF (Figure 1f).

The results of clinical studies examining human sera have demonstrated the indispensable role of RSV-neutralizing antibodies in preventing RSV infection [22,23]. We also found that the addition of the Gcc antigen could significantly enhance the NAb titers against RSV Long and B1 when compared to Ad5-PreF or Ad5-Gcc alone (Figure 4). This phenomenon may be caused by the additive effect between F and Gcc. During RSV infection, the G protein mainly plays an attachment role, and the F protein primarily mediates fusion. This finding further validates our hypothesis that Gcc acting in concert with PreF could elicit stronger virus neutralization. Additionally, we also detected weak neutralizing antibodies against RSV Long and RSV B1 in the FI-RSV immunization groups, which is consistent with the results of previous studies [24]. However, FI-RSV-immunized mice have been found to be unable to induce neutralizing antibodies in some studies in the literature [25,26].

A delicate balance between the Th17 and Treg cell subsets could play an important role in the pathogenesis of RSV infection. During RSV infection, the functions of both subsets are the opposite of one another regarding viral clearance and clinical severity. Th17 cells are known to promote inflammation, which can even result in autoimmunity. Treg cells attempt to control the pathology of VED in RSV infection [27,28]. The FI-RSV vaccinated mice showed enhanced recruitment of CD4 ^+^ T cells accompanied by the depletion of Treg cells from the lung, resulting in severe lung injury occurring upon RSV infection [29]. Durant et al. reported that Treg cells can prevent Th2 immune responses and pulmonary eosinophilia during respiratory syncytial virus infection in mice [30]. Our results also support this notion. The number of Treg cells in the FI-RSV vaccinated mice following RSV challenge significantly decreased compared with the mice challenged with PBS, and severe lung pathology was observed in their lung tissue. In contrast, the number of Treg cells in the mice vaccinated with Ad5-PreF and Ad5-Gcc significantly increased. Interestingly, Treg cells in the mice vaccinated with Ad5-PreF-Qa-Gcc significantly increased, and no significant difference was noted compared to the mice vaccinated with PBS (Figure 6b).

It is important to note that our study also has some limitations. Firstly, we only assessed the protective effect of the candidate vaccine in Balb/C mice whereas cotton rats, in contrast, are more sensitive to RSV infection and have a pathology more similar to that of humans [31,32]. Additionally, we only evaluated the candidate vaccine’s protective effect against the RSV Long strain but not the RSV B strain due to difficulties in reaching the required titer for Balb/C challenge (1 × 10^6^ PFU) in vitro. Furthermore, the inclusion of flexible and rigid peptides in the Gcc-foldon design may have additional immune effects on the body, which require further evaluation. Lastly, considering the fact that Gcc-foldon is a small antigen, we selected a relatively high dose of immunization according to literature reports to produce a high level of antibodies. In the future, we will examine in greater depth the relationship between dose and immune response.

In summary, the utilization of a PreF-Gcc antigen design strategy based on the Ad5 vector vaccine platform demonstrates a robust induction of both mucosal and systemic immune responses in Balb/c mice, effectively safeguarding against RSV challenge. The introduction of Gcc into the antigen design significantly enhances NAbs against both RSV Long and RSV B1 and enhances Treg cell responses, which is beneficial in preventing VED generation. Future studies will be conducted to evaluate the immunogenicity and protection offered by this vaccine candidate using the cotton rat model to provide more data support during preclinical studies.

## Figures and Tables

**Table 1:** Oligonucleotides in specific primers for the RT-PCR assay.

Primer	Nucleotides Sequence (5′-3′)
RSV Long q-PCR-F	GAACTCAGTGTAGGTAGAATGTTTGCA
RSV Long q-PCR-R	TTCAGCTATCATTTTCTCTGCCAAT
RSV Long probe	5′FAMATTTGAACCTGTCTGAACATTCCCGGTTGCAT 3′BHQ1

**Table 2:** Histopathological scores of lungs from immunized mice on day 5 following RSV challenge.

Inoculum	Alveolitis ^a^	Interstitial Pneumonia ^a^	Perivascular Bronchitis ^a^
PBS	0	0	0
Ad5-empty	1.6 ± 0.08	1.6 ± 0.15	2.25 ± 0.09
FI-RSV	2.12 ± 0.13	2.21 ± 0.12	2.9 ± 0.1
Ad5-PreF	0	0.6 ± 0.08	1.02 ± 0.05
Ad5-Gcc	0.82 ± 0.08	1.51 ± 0.05	1.51 ± 0.08
Ad5-PreF-Qa-Gcc	0	0.8 ± 0.12	1.15 ± 0.06

^a^ The severity scores were defined on a scale from 0 to 4 according to the H&E-stained sections as follows: 0—inflammation was not present, 1—minimal inflammation, 2—mild inflammation, 3—moderate inflammation, and 4—marked inflammation.

## Data Availability

All data related to this study are included in this article.

## References

[1] Rijsbergen L.C., Lamers M.M., Comvalius A.D., Koutstaal R.W., Schipper D., Duprex W.P., Haagmans B.L., de Vries R.D., de Swart R.L. (2021). Human Respiratory Syncytial Virus Subgroup A and B Infections in Nasal, Bronchial, Small-Airway, and Organoid-Derived Respiratory Cultures. mSphere.

[2] Freitas F.T.M., Pimentel C.C.P., Bianchini P.R., Carvalho R.M., Serafim A.P., Costa C.F.A. (2023). Evaluation of Severe Acute Respiratory Syndrome surveillance caused by respiratory viruses in a pediatric unit, 2013 to 2019. Rev. Paul. Pediatr..

[3] Tabor D.E., Fernandes F., Langedijk A.C., Wilkins D., Lebbink R.J., Tovchigrechko A., Ruzin A., Kragten-Tabatabaie L., Jin H., Esser M.T. (2020). Global Molecular Epidemiology of Respiratory Syncytial Virus from the 2017–2018 INFORM-RSV Study. J. Clin. Microbiol..

[4] Fuentes S., Coyle E.M., Beeler J., Golding H., Khurana S. (2016). Antigenic Fingerprinting following Primary RSV Infection in Young Children Identifies Novel Antigenic Sites and Reveals Unlinked Evolution of Human Antibody Repertoires to Fusion and Attachment Glycoproteins. PLoS Pathog..

[5] Fuentes S., Coyle E.M., Golding H., Khurana S. (2015). Nonglycosylated G-Protein Vaccine Protects against Homologous and Heterologous Respiratory Syncytial Virus (RSV) Challenge, while Glycosylated G Enhances RSV Lung Pathology and Cytokine Levels. J. Virol..

[6] Battles M.B., McLellan J.S. (2019). Respiratory syncytial virus entry and how to block it. Nat. Rev. Microbiol..

[7] Hendricks D.A., Baradaran K., McIntosh K., Patterson J.L. (1987). Appearance of a soluble form of the G protein of respiratory syncytial virus in fluids of infected cells. J. Gen. Virol..

[8] Chirkova T., Lin S., Oomens A.G.P., Gaston K.A., Boyoglu-Barnum S., Meng J., Stobart C.C., Cotton C.U., Hartert T.V., Moore M.L. (2015). CX3CR1 is an important surface molecule for respiratory syncytial virus infection in human airway epithelial cells. J. Gen. Virol..

[9] Johnson S.M., McNally B.A., Ioannidis I., Flano E., Teng M.N., Oomens A.G., Walsh E.E., Peeples M.E. (2015). Respiratory Syncytial Virus Uses CX3CR1 as a Receptor on Primary Human Airway Epithelial Cultures. PLoS Pathog..

[10] Zhu Y., Saribas A.S., Liu J., Lin Y., Bodnar B., Zhao R., Guo Q., Ting J., Wei Z., Ellis A. (2023). Protein expression/secretion boost by a novel unique 21-mer cis-regulatory motif (Exin21) via mRNA stabilization. Mol. Ther..

[11] Brakel K.A., Ma Y., Binjawadagi R., Harder O., Watts M., Li J., Binjawadagi B., Niewiesk S. (2022). Codon-optimization of the respiratory syncytial virus (RSV) G protein expressed in a vesicular stomatitis virus (VSV) vector improves immune responses in a cotton rat model. Virology.

[12] Krarup A., Truan D., Furmanova-Hollenstein P., Bogaert L., Bouchier P., Bisschop I.J.M., Widjojoatmodjo M.N., Zahn R., Schuitemaker H., McLellan J.S. (2015). A highly stable prefusion RSV F vaccine derived from structural analysis of the fusion mechanism. Nat. Commun..

[13] Rainho-Tomko J.N., Pavot V., Kishko M., Swanson K., Edwards D., Yoon H., Lanza L., Alamares-Sapuay J., Osei-Bonsu R., Mundle S.T. (2022). Immunogenicity and protective efficacy of RSV G central conserved domain vaccine with a prefusion nanoparticle. NPJ Vaccines.

[14] Liu J., Xu K., Xing M., Zhuo Y., Guo J., Du M., Wang Q., An Y., Li J., Gao P. (2021). Heterologous prime-boost immunizations with chimpanzee adenoviral vectors elicit potent and protective immunity against SARS-CoV-2 infection. Cell Discov..

[15] Murphy B.R., Walsh E.E. (1988). Formalin-inactivated respiratory syncytial virus vaccine induces antibodies to the fusion glycoprotein that are deficient in fusion-inhibiting activity. J. Clin. Microbiol..

[16] Kwon Y.M., Lee Y., Kim K.H., Jung Y.J., Li Z., Jeeva S., Lee S., Moore M.L., Kang S.M. (2019). Antigenicity and immunogenicity of unique prefusion-mimic F proteins presented on enveloped virus-like particles. Vaccine.

[17] Luo J., Qin H., Lei L., Lou W., Li R., Pan Z. (2022). Virus-like particles containing a prefusion-stabilized F protein induce a balanced immune response and confer protection against respiratory syncytial virus infection in mice. Front. Immunol..

[18] Greinacher A., Thiele T., Warkentin T.E., Weisser K., Kyrle P.A., Eichinger S. (2021). Thrombotic Thrombocytopenia after ChAdOx1 nCov-19 Vaccination. N. Engl. J. Med..

[19] Hwang J., Lee S.B., Lee S.W., Lee M.H., Koyanagi A., Jacob L., Tizaoui K., Yon D.K., Shin J.I., Smith L. (2021). Comparison of vaccine-induced thrombotic events between ChAdOx1 nCoV-19 and Ad26.COV.2.S vaccines. J. Autoimmun..

[20] Afkhami S., D’Agostino M.R., Zhang A., Stacey H.D., Marzok A., Kang A., Singh R., Bavananthasivam J., Ye G., Luo X. (2022). Respiratory mucosal delivery of next-generation COVID-19 vaccine provides robust protection against both ancestral and variant strains of SARS-CoV-2. Cell.

[21] Mekseepralard C., Toms G.L., Routledge E.G. (2006). Protection of mice against Human respiratory syncytial virus by wild-type and aglycosyl mouse-human chimaeric IgG antibodies to subgroup-conserved epitopes on the G glycoprotein. J. Gen. Virol..

[22] Walsh E.E., Pérez Marc G., Zareba A.M., Falsey A.R., Jiang Q., Patton M., Polack F.P., Llapur C., Doreski P.A., Ilangovan K. (2023). Efficacy and Safety of a Bivalent RSV Prefusion F Vaccine in Older Adults. N. Engl. J. Med..

[23] Falsey A.R., Williams K., Gymnopoulou E., Bart S., Ervin J., Bastian A.R., Menten J., De Paepe E., Vandenberghe S., Chan E.K.H. (2023). Efficacy and Safety of an Ad26.RSV.preF-RSV preF Protein Vaccine in Older Adults. N. Engl. J. Med..

[24] Hwang H.S., Kwon Y.M., Lee J.S., Yoo S.E., Lee Y.N., Ko E.J., Kim M.C., Cho M.K., Lee Y.T., Jung Y.J. (2014). Co-immunization with virus-like particle and DNA vaccines induces protection against respiratory syncytial virus infection and bronchiolitis. Antiviral Res..

[25] van der Fits L., Bolder R., Heemskerk-van der Meer M., Drijver J., van Polanen Y., Serroyen J., Langedijk J.P.M., Schuitemaker H., Saeland E., Zahn R. (2020). Adenovector 26 encoded prefusion conformation stabilized RSV-F protein induces long-lasting Th1-biased immunity in neonatal mice. NPJ Vaccines.

[26] Joyce C., Scallan C.D., Mateo R., Belshe R.B., Tucker S.N., Moore A.C. (2018). Orally administered adenoviral-based vaccine induces respiratory mucosal memory and protection against RSV infection in cotton rats. Vaccine.

[27] Ruckwardt T.J., Bonaparte K.L., Nason M.C., Graham B.S. (2009). Regulatory T cells promote early influx of CD8+ T cells in the lungs of respiratory syncytial virus-infected mice and diminish immunodominance disparities. J. Virol..

[28] Fulton R.B., Meyerholz D.K., Varga S.M. (2010). Foxp3+ CD4 regulatory T cells limit pulmonary immunopathology by modulating the CD8 T cell response during respiratory syncytial virus infection. J. Immunol..

[29] Loebbermann J., Durant L., Thornton H., Johansson C., Openshaw P.J. (2013). Defective immunoregulation in RSV vaccine-augmented viral lung disease restored by selective chemoattraction of regulatory T cells. Proc. Natl. Acad. Sci. USA.

[30] Durant L.R., Makris S., Voorburg C.M., Loebbermann J., Johansson C., Openshaw P.J. (2013). Regulatory T cells prevent Th2 immune responses and pulmonary eosinophilia during respiratory syncytial virus infection in mice. J. Virol..

[31] Boukhvalova M.S., Prince G.A., Blanco J.C. (2009). The cotton rat model of respiratory viral infections. Biologicals.

[32] Cox F., Saeland E., Thoma A., van den Hoogen W., Tettero L., Drijver J., Vaneman C., van Polanen Y., Ritschel T., Bastian A.R. (2023). RSV A2-Based Prefusion F Vaccine Candidates Induce RSV A and RSV B Cross Binding and Neutralizing Antibodies and Provide Protection against RSV A and RSV B Challenge in Preclinical Models. Vaccines.

